# Social influences on smoking cessation in mid-life: Prospective cohort of UK women

**DOI:** 10.1371/journal.pone.0226019

**Published:** 2019-12-06

**Authors:** Jaime L. Martin, Isobel Barnes, Jane Green, Gillian K. Reeves, Valerie Beral, Sarah Floud

**Affiliations:** 1 Faculty of Medicine, Dentistry and Health Sciences, Melbourne University, Melbourne, Victoria, Australia; 2 Cancer Epidemiology Unit, Nuffield Department of Population Health, University of Oxford, Oxford, United Kindom; Ruhr University Bochum, GERMANY

## Abstract

**Introduction:**

Decisions to quit smoking are thought to be influenced by social factors such as friends, family and social groups, but there have been few attempts to examine comprehensively the influence of a range of social factors on smoking cessation. In the largest study to date, we examined whether smoking cessation was associated with marital status and the smoking habits of a partner, socio-economic status and social participation.

**Methods:**

In the prospective Million Women Study, 53,650 current smokers in 2001 (mean age 58.3, SD 4.4) reported their smoking status 4 years later; and reported on social factors on both occasions. Logistic regression yielded odds ratios (ORs) and 99% confidence intervals (CIs) for stopping smoking in the next 4 years by marital status, whether their partner smoked, deprivation, education, and participation in social activities.

**Results:**

31% (16,692) of the current smokers at baseline had stopped after 4 years. Smokers who were partnered at baseline were more likely to quit than those who were not partnered (OR 1.13, 99% CI 1.06–1.19). Compared to having a partner who smoked throughout, those who had a non-smoking partner throughout were more likely to quit (OR 2.01, 99% CI 1.86–2.17), and those who had a partner who smoked at baseline but stopped smoking in the next 4 years were even more likely to quit (OR 6.00, 5.41–6.67). There was no association with cessation for education or deprivation. The association with social participation varied by type of activity but was null overall.

**Conclusion:**

Women who were partnered were most likely to stop smoking if their partner also stopped smoking. There was little evidence of a strong influence of either socio-economic status or social participation on smoking cessation. These results emphasise the importance of a spouse’s smoking habits on the likelihood of a smoker successfully quitting smoking.

## Introduction

There are more than one billion smokers worldwide.[1] Within high income countries, such as the UK, smoking rates are declining.[2] Nevertheless in 2016 16% of people in the UK smoked (18% of men and 14% of women), which equates to approximately 7.6 million people, and smoking remains a leading cause of preventable deaths in the UK.[2] Quitting smoking much reduces the risk of premature death, with smoking cessation in middle age avoiding two-thirds of the excess mortality related to smoking.[3–7]

Smoking rates tend to be higher among those who are not married or living with a partner, those with lower socio-economic status, and those who are socially isolated.[8–13] Therefore it is important to understand whether these factors influence smoking cessation. Previous research on the role of marital status suggests that being married or living with a partner leads to a higher likelihood of quitting smoking [14, 15] and that having a non-smoking partner also increases the chances of quitting,[16] although this has not always been found to be of benefit to women.[17] There is also some suggestive evidence that having a partner who quits smoking may have a greater influence over smoking cessation than having a partner who is a non-smoker.[15, 18] Previous research examining the influence of socioeconomic status on smoking cessation suggests that there may be a social gradient in the success of attempts to quit smoking and that women of lower socio-economic status may be less successful than men in quitting.[11, 16] It has also been suggested that participation in social activities might have a beneficial effect on smoking cessation, but the associations are weak.[19]

Using data from a large prospective study of UK women with repeated measures of smoking status, we aimed to compare the associations between a range of social factors and smoking cessation among women in the largest sample of smokers to date. Specifically, the aim of this analysis was to examine the association between marital status and smoking cessation, and, for married or partnered women, to assess the association between their partner’s smoking habits and their odds of quitting smoking. In addition, we examined the association between socio-economic status and smoking cessation using two measures of socio-economic status, area deprivation and educational qualifications; and the association between participation in a range of different social activities and smoking cessation.

## Methods

### Participants

This analysis included women in the population-based prospective Million Women Study. Details of the study have been described elsewhere.[20] Briefly, 1.3 million women (aged 50–64 years) were recruited in 1996–2001 through the UK National Breast Screening Programme and answered a paper questionnaire on socio-demographic, anthropometric and behavioural factors. After an average of three years, all surviving participants in the cohort were resurveyed using a postal questionnaire, with a response rate of 65%. At this point, study participants were asked for the first time about their marital status, the smoking habits of their husband/partner and participation in social activities. The date of completion of this questionnaire, in 2001 on average, is therefore the baseline for this analysis. A further postal questionnaire was sent to all surviving participants about 4 years later and is the follow-up point for this analysis. The relevant study questionnaires can be viewed on the study website (www.millionwomenstudy.org; see “3 year re-survey” and “8 year re-survey”), as well as further details of the data and access policies. Respondents gave written informed consent to follow-up and ethical approval was provided by the Oxford and Anglia Multi-Centre Research Ethics Committee.

### Smoking

This analysis was conducted only in women who replied “yes” to the question “Are you a smoker now?” at baseline and who completed a questionnaire 4 years later. Other questions on smoking that were asked were: the age women started smoking regularly and the number of cigarettes currently smoked on average per day. Four years later, the participants in this analysis who responded “no” to the question “Are you a smoker now?” were defined as ‘quitters’, and “yes” as ‘continuing smokers’.

### Social influences

Marital status was defined according to responses to the following question at baseline: “Are you currently married or living with a partner?” Those who replied “yes” are referred to, henceforth, as ‘partnered’, while those who responded “no” are referred to as ‘unpartnered’. The latter includes women who were never married, as well as women who were divorced, separated or widowed.[10] Partnered women were also asked whether their partner smoked. Both questions were repeated four years later.

An area-based measure of deprivation was estimated for each participant using reported postcode at study recruitment within the smallest geographical unit to which a Townsend score [21] could be assigned (census enumeration districts in England, census output areas in Scotland) and was categorised by tertiles before any exclusions were made for this analysis. The Townsend index is constructed from four census variables: percentage of households without a car, percentage of overcrowded households, percentage of households not owner-occupied and percentage of persons unemployed. Level of education was also reported at study recruitment, and divided into four categories: tertiary qualifications (college or university), secondary qualifications (A levels or O levels usually obtained at 18 and 16 years old respectively), technical qualifications (nursing, teaching, clerical or commercial), no educational qualifications.

Participation in social activities was assessed by asking women at baseline if they belonged to or participated in any of the following: ‘religious group’, ‘voluntary work’, ‘adult education’, ‘art/craft group’, ‘dancing group’, ‘sports club’, ‘yoga’, ‘music/singing group’ and ‘bingo’. ‘Art/craft group’ and ‘music/singing group’ were combined into one variable called ‘art/craft/music’ and similarly ‘sports club’ and ‘yoga’ were combined together as ‘sports/yoga’ due to the similar characteristics of women participating in these activities. ‘Any participation’ was defined as participation in any one of the listed activities. There was also an option to answer ‘other group’ in the questionnaire but this was not analysed because this category could encompass a wide range of different types of activity.

### Statistical analysis

Of the 95,346 participants who reported being current smokers at baseline, 41,696 of them did not complete a questionnaire four years later and so were not included in the analysis. The analysis was conducted in study participants who reported being current smokers at baseline and who replied to the study questionnaire 4 years later (n = 57,431). Participants were then excluded if they had missing information on the exposures of interest (n = 2860); if they had not been asked about participation in bingo, as it was not included as an option in the first wave of questionnaires (n = 709); or if they provided inconsistent responses (e.g. they said they were not partnered but also that their partner was a smoker, n = 212). This left 53,650 current smokers in the analyses.

Logistic regression yielded odds ratios and 99% confidence intervals for stopping smoking between baseline and four years later in relation to the following social factors: marital status at baseline, deprivation, education and social participation. With regard to marital status, two further analyses were undertaken. First, the association with change in marital status was investigated, with those who were unpartnered at both time points as the reference. Second, in women who reported being partnered at baseline and 4 years later, we compared the odds of stopping smoking in women who had a partner who was a smoker at both time points with the odds of stopping smoking in women who had a partner who was a non-smoker at both time points, or who had a partner who stopped smoking during the 4 year period, or who had a partner who started smoking during the 4 year period.

We adjusted for the following factors a priori: age at baseline (<55, 55–59, ≥60 years), age of smoking initiation (<16, 16–19, 20+ years), average number of cigarettes smoked per day (<10, 10–14, 15–20, 20+ cigarettes), time between the resurveys (<3, 3, ≥4 years) because women with a longer period of follow-up had more opportunity to quit, and self-rated health (poor, fair, good, excellent) because ill-health might lead to quitting smoking. Missing data for the adjustment variables (<0.8% for each variable) were assigned to a separate category. Associations with marital status at baseline, education, deprivation, partner’s smoking status, and social participation were mutually adjusted, using ‘any activity’ to represent social participation. The associations with each specific activity were not adjusted for participation in the other activities. There was no difference in the results when associations with education and deprivation were not mutually adjusted, so these socio-economic variables were included together.

It has been shown that being diagnosed with a smoking-related disease is a reason for stopping smoking,[22] so two sensitivity analyses were conducted, firstly adjusting for previous hospital admission for major diseases (heart disease, stroke, cancer, chronic obstructive airways disease) and secondly, excluding participants who had had a previous hospital admission for these major diseases.

All analyses used Stata 15.1 (StataCorp., College Station, TX, USA).

## Results

53,650 women who reported being current smokers at baseline were included in the analysis; 31% (16,692) of whom had stopped smoking four years later. At baseline, the women included in the analysis reported smoking 15 cigarettes per day, on average, and had started smoking at 19 years old (Table 1). Their mean age was 58.3 (SD 4.4) years, 46% had no educational qualifications, 72% were partnered, and 46% participated in at least one social activity. There were some differences in baseline characteristics between those who had stopped smoking four years later and those who continued to smoke: those who quit smoking smoked fewer cigarettes per day and were slightly less likely to rate their health as poor or fair. Compared with the women in the analysis group, current smokers at baseline who did not complete a further questionnaire 4 years later (and so could not be included in this analysis) tended to be slightly more deprived and slightly less likely to have educational qualifications or to participate in any of the social activities (S1 Table).

**Table 1:** Baseline characteristics of current smokers included in the analysis.

	All current smokers at baseline		Current smokers who ceased smoking in next 4 years		Current smokers who continued smoking over next 4 years	
N	53,650		16,692		36,958	
Age, mean (SD)	58.3	(4.4)	58.3	(4.4)	58.4	(4.5)
No. cigarettes smoked/day, mean (SD)*	14.9	(7.2)	13.3	(7.2)	15.7	(7.2)
Age started smoking, mean years (SD)*	19.0	(5.9)	19.4	(5.9)	18.8	(5.1)
Most deprived tertile, %(n)	30.9	(16,570)	28.9	(4,827)	31.8	(11,743)
No educational qualifications, %(n)	45.7	(24,537)	44.7	(7,457)	46.2	(17,080)
Partnered, %(n)	72	(38,650)	74.6	(12,449)	70.9	(26,201)
Participate in religious group, %(n)	7.7	(4,152)	8.5	(1,413)	7.4	(2,739)
Participate in voluntary work, %(n)	12.7	(6,803)	12.6	(2,107)	12.7	(4,696)
Participate in adult education, %(n)	8.0	(4,303)	8.9	(1,480)	7.6	(2,823)
Participate in art/craft/music, %(n)	7.0	(3,741)	7.9	(1,318)	6.6	(2,423)
Participate in dancing, %(n)	4.7	(2,539)	5.1	(846)	4.6	(1,693)
Participate in sports/yoga, %(n)	15.2	(8,134)	17.4	(2,901)	14.2	(5,233)
Participate in bingo, %(n)	12.2	(6,526)	10.3	(1,714)	13.0	(4,812)
Participate in any activity, %(n)	46.4	(24,867)	47.5	(7,924)	45.8	(16,943)
Poor/fair self-rated health, %(n)*	31.1	(16,185)	29.1	(4,719)	31.9	(11,466)
Previous serious illness, %(n)*†*	7.2	(3879)	6.2	(1039)	7.7	(2840)

* percentage missing for these variables: no. cigarettes smoked/day (0.8%), age started smoking (0.7%), self-rated health (2.9%)

†hospital admission for heart disease, stroke, cancer or chronic obstructive airways disease

In adjusted analyses, women who were partnered at baseline were more likely to have quit smoking four years later (OR 1.13, 99% CI 1.06–1.19 for stopping smoking in women who were partnered vs not partnered at baseline) (Table 2). When the association with marital status was looked at over the period of the study, those who were partnered at both time points were more likely to quit than those who were unpartnered at both time points (OR = 1.18, 1.11–1.25) (S2 Table). Those who were partnered at baseline and not partnered 4 years later were no different from those who were unpartnered throughout (OR = 0.93, 0.83–1.04), whereas the small proportion of women who were not partnered at baseline and were partnered 4 years later were more likely to have quit smoking (OR = 1.34, 1.07–1.68).

**Table 2:** Odds ratios (99% confidence intervals) for smoking cessation in relation to various social factors at baseline.

Social Factors at baseline	No. of current smokers	No. who ceased smoking in next 4 years	Unadjusted OR (99% CI)		Adjusted OR (99% CI)	
**Marital status**						
Not partnered	15,000	4,243	1.00		1.00	
Partnered	38,650	12,449	1.20	(1.14,1.27)	1.13	(1.06,1.19)
**Education**						
Tertiary	6,114	2,032	1.00		1.00	
Secondary	14,385	4,476	0.91	(0.83,0.99)	0.93	(0.85,1.01)
Technical	8,614	2,727	0.93	(0.85,1.02)	0.97	(0.88,1.06)
No qualifications	24,537	7,457	0.88	(0.81,0.95)	0.95	(0.88,1.03)
**Deprivation**						
Least deprived tertile	19,082	6,270	1.00		1.00	
Middle deprivation tertile	17,998	5,595	0.92	(0.87,0.98)	0.97	(0.92,1.03)
Most deprived tertile	16,570	4,827	0.84	(0.79,0.89)	0.95	(0.89,1.01)
**Social participation**						
Not in religious group	49,498	15,279	1.00		1.00	
Religious group	4,152	1,413	1.16	(1.06,1.26)	1.05	(0.96,1.15)
Not doing voluntary work	46,847	14,585	1.00		1.00	
Voluntary work	6,803	2,107	0.99	(0.92,1.07)	0.92	(0.85,0.99)
Not in adult education	49,347	15,212	1.00		1.00	
Adult education	4,303	1,480	1.18	(1.08,1.28)	1.10	(1.00,1.20)
Not doing art/craft/music	49,909	15,374	1.00		1.00	
Art/craft/music	3,741	1,318	1.22	(1.11,1.34)	1.13	(1.03,1.25)
Not dancing	51,111	15,846	1.00		1.00	
Dancing	2,539	846	1.11	(1.00,1.24)	1.04	(0.93,1.16)
Not doing sports/yoga	45,516	13,791	1.00		1.00	
Sports/yoga	8,134	2,901	1.28	(1.19,1.36)	1.12	(1.05,1.20)
Not doing bingo	47,124	14,978	1.00		1.00	
Bingo	6,526	1,714	0.76	(0.71,0.83)	0.84	(0.78,0.91)
Not participating in any activity	28,783	8,768	1.00		1.00	
Any activity	24,867	7,924	1.07	(1.02,1.12)	1.01	(0.96,1.06)

Adjusted for age, age of smoking initiation, average number of cigarettes smoked per day, time between surveys, and self-rated health. Associations with education, deprivation, marital status and social participation were mutually adjusted, using ‘any activity’ to represent social participation. The associations with each specific activity were not adjusted for participation in the other activities.

**Fig 1:**
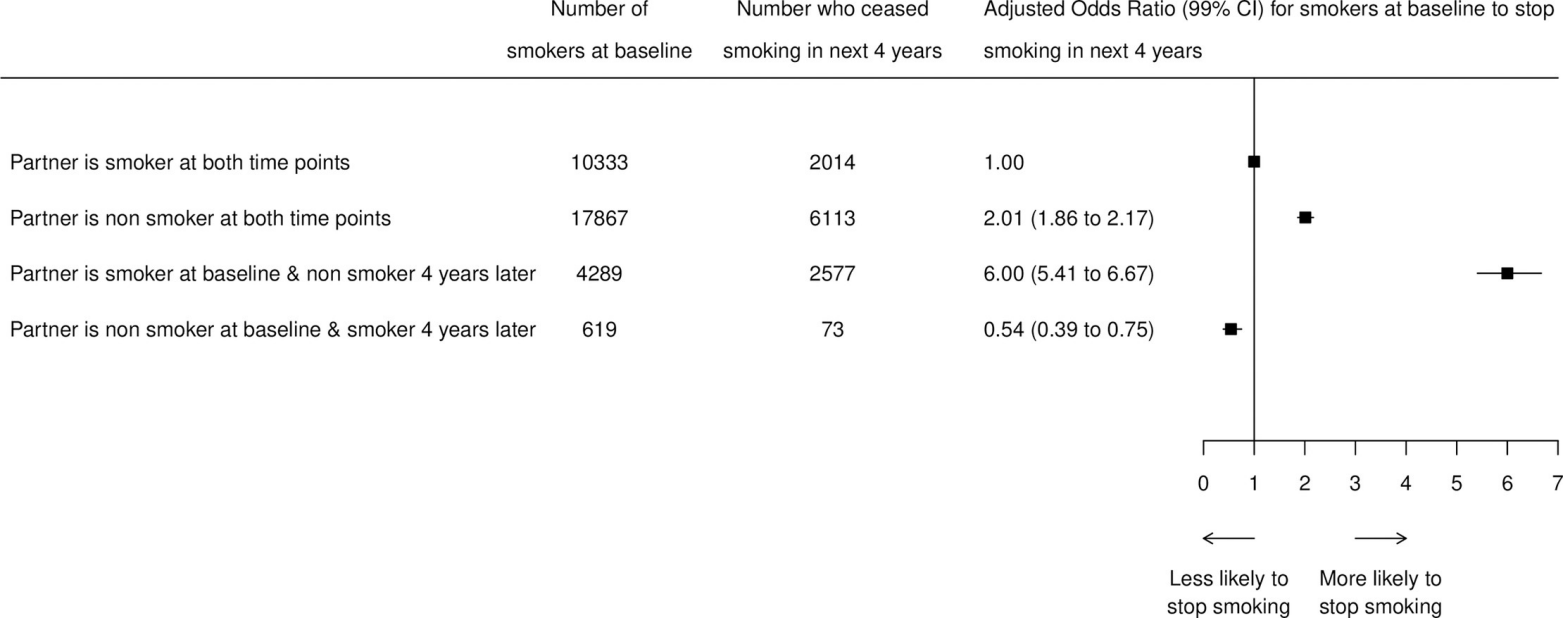
Odds ratios (99% confidence intervals) for smoking cessation in current smokers who have partner at both time points. Adjusted for age at baseline, age of smoking initiation, average number of cigarettes smoked per day, time between surveys, self-rated health, education, deprivation, and social participation.

Of the 33,108 women who were partnered at baseline, 44% had a partner who also smoked at baseline (Fig 1). Compared to women who had a partner who was a smoker at both time points, having a partner who was a non-smoker throughout was associated with a doubling of the odds of stopping smoking (OR 2.01, 99% CI 1.86–2.17), and having a partner who smoked initially but stopped in the next 4 years was associated with a 6-fold increase in odds of stopping smoking (OR 6.00, 99% CI 5.41–6.67). However, having a partner who started smoking in the next 4 years was associated with lower odds of stopping smoking (OR 0.54, 99% CI 0.39–0.75).

The likelihood of smoking cessation was not associated with deprivation (OR 0.95, 99% CI 0.89–1.01 for stopping smoking in most vs least deprived third) nor with educational attainment (OR 0.95, 99% CI 0.88–1.04 for stopping smoking in women with no educational qualifications vs tertiary qualifications) (Table 2).

Smoking cessation was also not associated with social participation in general (OR 1.01, 99% CI 0.96–1.06 for stopping smoking in women who participate in any of the list of social activities vs not; Table 2). However, participation in art/craft/music groups (OR 1.13, 99% CI 1.03–1.25) or in sports club/yoga (OR 1.12, 99% CI 1.05–1.20) was associated with a significantly increased likelihood of quitting, whereas participation in bingo was associated with significantly decreased odds of quitting (OR 0.84, 99% CI 0.78‒0.91).

For each analysis shown in Table 2, adjustment for age, age of smoking initiation, average number of cigarettes smoked per day, time between surveys, self-rated health and the other social factors had only a small impact on the strength of the associations, suggesting that these results are robust to confounding. Sensitivity analyses adjusting for hospital admissions for major diseases at baseline and alternatively excluding those who had a major disease at baseline had no impact on the results (S3 and S4 Tables).

## Discussion

In the largest study to date of the social influences on smoking cessation, women who smoked were more likely to quit smoking if they were married or living with a partner, and particularly if their partner had stopped smoking during the follow-up period. After adjustment for confounding factors, neither deprivation nor education was significantly associated with a greater likelihood of smoking cessation. The association with social participation was null, although there was some suggestion of variation by type of social activity.

Our finding of the positive influence of being married or partnered on smoking cessation confirms the findings from a study of approximately 5000 current smokers in a Swedish longitudinal database, which found that women were more likely to quit smoking if they were married or if they got married during the study than those who were unmarried or who separated or got divorced.[14]

When we concentrated the analysis on only those who were partnered throughout the study, we found that, if participants had a partner who was a non-smoker throughout, they were more likely to give up smoking than those who had a partner who smoked throughout. This concurs with results from a study which showed that having a spouse who was a non-smoker was related to a greater likelihood of stopping smoking compared to having a spouse who was a smoker.[16] We also found that if the participant’s partner gave up smoking during the study, then the participant had 6 fold higher odds of giving up smoking themselves than if their partner smoked throughout, and therefore this was even more beneficial than having a partner who was a non-smoker throughout. An analysis of data on 1005 smokers from the English Longitudinal Study of Ageing found that having a partner who also quit smoking was more strongly associated with quitting than having a non-smoking partner, but their estimates were based on only 30 smokers whose partners quit and so the magnitude of the association was not clear (OR 11.23, 95% CI 4.58–27.52).[18] In an analysis from the US Health and Retirement study amongst 5250 smokers, the only significant positive association with smoking cessation was having a partner who also quit smoking; all other associations were not statistically significant.[15] Thus our study provides clearer evidence from a much larger sample than has previously been presented on the influential role of a partner’s smoking behaviour on the likelihood of smoking cessation.

It has been suggested that increased deprivation may reduce the likelihood of successful cessation due to a lack of support, lower adherence to treatment or life stress;[11] and there is some evidence to suggest that people with lower socioeconomic status tend to smoke more cigarettes per day and are therefore more nicotine dependent.[23] Other studies have found a statistically significant lower likelihood of cessation in more deprived participants,[16] but did not adjust for number of cigarettes or age started smoking. In the present analysis however, after adjustment for number of cigarettes and the age the women started smoking, the association between socio-economic status and smoking cessation was not statistically significant. Despite this, there was a lower likelihood of quitting for those who played bingo, which is a marker of deprivation in this cohort.[9]

Our results showed a weak association between social participation generally and smoking cessation. There was some variation by type of activity, with lower odds of quitting if the favoured activity was bingo. There were also greater odds of quitting smoking in those who participated in sporting activities or art/craft activities, even after adjustment for deprivation and education which might influence the opportunity to participate in these activities. It is hypothesised that through engagement with others, social participation will provide opportunities to receive social support and to access other people’s knowledge, values and norms.[24] This might in turn challenge perceptions of acceptable behaviours including smoking, through exposure to alternative viewpoints.[24]

The strength of this population-based study is its large size with information at more than one time-point on a large number of smokers and on the smoking habits of the partners of those smokers. After four years, about a third of the women had stopped smoking, which is consistent with national quitting rates at that time.[25] We were able to look at a number of factors in one study and have confirmed that for older women, the main factor influencing cessation is the smoking behaviour of their partner. This indicates that the influences on smoking cessation in an older cohort may be different from a younger cohort. In addition, whilst many studies have looked at the effect of a smoking/non-smoking partner, fewer have looked more dynamically at the effect of a partner quitting or taking up smoking across the study period. [15, 18] We were also able to adjust for self-rated health to account for the increased likelihood of quitting due to ill-health.[26–28] A limitation is that some participants who were smokers at baseline but did not return the follow-up questionnaire (and so were not included in the analysis) were more likely to be deprived and have fewer educational qualifications, and so the null association between the measures of socio-economic status and smoking cessation found here may be an underestimate. A further limitation is that the data on social participation were collected prior to the prohibition of smoking in all public areas in the UK in 2007. As a result, smoking behaviours relating to social activities may since have changed.

## Conclusions

Given the known health benefits of stopping smoking, it is important to recognise the impact of social influences on an individual’s decision to stop smoking. The results of this study emphasise that smoking cessation is strongly influenced by the smoking behaviour of spouses and partners.

## Supporting information

S1 Table Baseline characteristics of current smokers included in analyses and of current smokers excluded from analyses. (DOCX)

S2 Table Odds ratios (99% confidence intervals) for smoking cessation in relation to marital status over the period of study. (DOCX)

S3 Table Sensitivity analysis for Table 2, showing results adjusting for previous serious illness (hospital admission for heart disease, stroke, cancer, chronic obstructive airways disease) rather than self-rated health, and results after excluding women with previous serious illness. (DOCX)

S4 Table Sensitivity analysis for Fig 1, showing results adjusting for previous serious illness (hospital admission for heart disease, stroke, cancer, chronic obstructive airways disease) rather than self-rated health, and results after excluding women with previous serious illness. (DOCX)
