# Supplementary material for: Social influences on smoking cessation in mid-life: Prospective cohort of UK women
Source: PLoS One. 2019 Dec 6;14(12):e0226019. doi: 10.1371/journal.pone.0226019 (PMC6897408; doi:10.1371/journal.pone.0226019)
Supplement: S1 Table — (DOCX) [file pone.0226019.s001.docx]

**S1 Table** **Baseline characteristics of current smokers included in analyses and of current smokers excluded from analyses**

|  | **Current smokers at baseline included in analysis** | |  | **Current smokers at baseline who did not return survey 4 years later** | |
| --- | --- | --- | --- | --- | --- |
| N | 53,650 |  |  | 41,696 |  |
| Age, mean (SD) | 58.3 | ( 4.4) |  | 58.3 | ( 4.5) |
| No. of cigarettes smoked per day, mean (SD) | 14.9 | ( 7.3) |  | 14.9 | ( 7.2) |
| Age started smoking, mean years (SD) | 19.0 | ( 5.4) |  | 19.0 | ( 5.5) |
| Most deprived tertile, %(n) | 30.9 | (16,570) |  | 35.5 | (14,820) |
| No educational qualifications, %(n) | 45.7 | (24,537) |  | 57.4 | (23,925) |
| Partnered, %(n) | 72.0 | (38,650) |  | 73.5 | (30,633) |
| Participate in religious group, %(n) | 7.7 | ( 4,152) |  | 5.6 | ( 2,318) |
| Participate in voluntary work, %(n) | 12.7 | ( 6,803) |  | 9.3 | ( 3,892) |
| Participate in adult education, %(n) | 8.0 | ( 4,303) |  | 5.3 | ( 2,227) |
| Participate in art/craft/music group, %(n) | 7.0 | ( 3,741) |  | 5.1 | ( 2,116) |
| Participate in dancing group, %(n) | 4.7 | ( 2,539) |  | 4.2 | ( 1,747) |
| Participate in sports club/yoga, %(n) | 15.2 | ( 8,134) |  | 11.7 | ( 4,880) |
| Participate in bingo, %(n) | 12.2 | ( 6,526) |  | 15.6 | ( 6,503) |
| Participate in any activity, %(n) | 46.4 | (24,867) |  | 41.6 | (17,354) |
| Poor/fair self-rated health, %(n) | 31.1 | (16,185) |  | 37.6 | (15,135) |
