# Supplementary material for: Social influences on smoking cessation in mid-life: Prospective cohort of UK women
Source: PLoS One. 2019 Dec 6;14(12):e0226019. doi: 10.1371/journal.pone.0226019 (PMC6897408; doi:10.1371/journal.pone.0226019)
Supplement: S2 Table — (DOCX) [file pone.0226019.s002.docx]

**S2 Table. Odds ratios (99% confidence intervals) for smoking cessation in relation to marital status over the period of study.**

|  | **No. of current smokers** | **No. who ceased smoking in next 4 years** |  | **Adjusted OR (99%CI)** | |
| --- | --- | --- | --- | --- | --- |
|  |  |  |  |  |  |
| Not partnered at both time points | 13,332 | 3,709 |  | 1.00 |  |
| Partnered at both time points | 34,981 | 11,409 |  | 1.18 | (1.11,1.25) |
| Partnered at baseline and not partnered 4 years later | 3,211 | 909 |  | 0.93 | (0.83,1.04) |
| Not partnered at baseline and partnered 4 years later | 648 | 225 |  | 1.34 | (1.07,1.68) |

Adjusted for age at baseline, age of smoking initiation, average number of cigarettes smoked per day, time between surveys, self-rated health, education, deprivation, and social participation.
