# Supplementary material for: Social influences on smoking cessation in mid-life: Prospective cohort of UK women
Source: PLoS One. 2019 Dec 6;14(12):e0226019. doi: 10.1371/journal.pone.0226019 (PMC6897408; doi:10.1371/journal.pone.0226019)
Supplement: S3 Table — (DOCX) [file pone.0226019.s003.docx]

# S3 Table. Sensitivity analysis for Table 2, showing results adjusting for previous serious illness (hospital admission for heart disease, stroke, cancer, chronic obstructive airways disease) rather than self-rated health, and results after excluding women with previous serious illness

|  | **Adjusted for previous serious illness** | | | |  | **Excluding previous serious illness** | | | |
| --- | --- | --- | --- | --- | --- | --- | --- | --- | --- |
| **Social factors at baseline** | **No. of current smokers** | **No. who ceased smoking in next 4 years** | **OR** | **99% CI** |  | **No. of current smokers** | **No. who ceased smoking in next 4 years** | **OR** | **99% CI** |
| **Marital Status** |  |  |  |  |  |  |  |  |  |
| Not partnered | 15,000 | 4,243 | 1.00 |  |  | 13,672 | 3,938 | 1.00 |  |
| Partnered | 38,650 | 12,449 | 1.13 | (1.06,1.19) |  | 36,099 | 11,715 | 1.12 | (1.05,1.18) |
|  |  |  |  |  |  |  |  |  |  |
| **Education** |  |  |  |  |  |  |  |  |  |
| Tertiary | 6,114 | 2,032 | 1.00 |  |  | 5,731 | 1,919 | 1.00 |  |
| Secondary | 14,385 | 4,476 | 0.93 | (0.85,1.01) |  | 13,461 | 4,216 | 0.93 | (0.85,1.01) |
| Technical | 8,614 | 2,727 | 0.97 | (0.88,1.06) |  | 8,008 | 2,575 | 0.98 | (0.89,1.08) |
| No qualifications | 24,537 | 7,457 | 0.95 | (0.87,1.03) |  | 22,571 | 6,943 | 0.95 | (0.87,1.04) |
|  |  |  |  |  |  |  |  |  |  |
| **Deprivation** |  |  |  |  |  |  |  |  |  |
| Least deprived tertile | 19,082 | 6,270 | 1.00 |  |  | 17,891 | 5,910 | 1.00 |  |
| Middle deprived tertile | 17,998 | 5,595 | 0.97 | (0.92,1.03) |  | 16,744 | 5,258 | 0.98 | (0.92,1.04) |
| Most deprived tertile | 16,570 | 4,827 | 0.95 | (0.89,1.01) |  | 15,136 | 4,485 | 0.96 | (0.90,1.03) |
|  |  |  |  |  |  |  |  |  |  |
| **Social Participation** |  |  |  |  |  |  |  |  |  |
| Not in religious group | 49,498 | 15,279 | 1.00 |  |  | 45,933 | 14,327 | 1.00 |  |
| Religious group | 4,152 | 1,413 | 1.05 | (0.96,1.16) |  | 3,838 | 1,326 | 1.06 | (0.96,1.16) |
| Not in voluntary group | 46,847 | 14,585 | 1.00 |  |  | 43,449 | 13,665 | 1.00 |  |
| Voluntary group | 6,803 | 2,107 | 0.92 | (0.85,0.99) |  | 6,322 | 1,988 | 0.93 | (0.86,1.00) |
| Not in adult education | 49,347 | 15,212 | 1.00 |  |  | 45,732 | 14,253 | 1.00 |  |
| Adult education | 4,303 | 1,480 | 1.10 | (1.00,1.20) |  | 4,039 | 1,400 | 1.09 | (1.00,1.20) |
| Not doing art/craft/music | 49,909 | 15,374 | 1.00 |  |  | 46,297 | 14,418 | 1.00 |  |
| Art/craft/music | 3,741 | 1,318 | 1.13 | (1.03,1.25) |  | 3,474 | 1,235 | 1.13 | (1.03,1.25) |
| Not dancing | 51,111 | 15,846 | 1.00 |  |  | 47,395 | 14,845 | 1.00 |  |
| Dancing | 2,539 | 846 | 1.04 | (0.93,1.16) |  | 2,376 | 808 | 1.06 | (0.94,1.19) |
| Not doing sports/yoga | 45,516 | 13,791 | 1.00 |  |  | 42,122 | 12,908 | 1.00 |  |
| Sports/yoga | 8,134 | 2,901 | 1.12 | (1.05,1.20) |  | 7,649 | 2,745 | 1.12 | (1.04,1.20) |
| Not doing bingo | 47,124 | 14,978 | 1.00 |  |  | 43,861 | 14,080 | 1.00 |  |
| Bingo | 6,526 | 1,714 | 0.84 | (0.78,0.91) |  | 5,910 | 1,573 | 0.84 | (0.77,0.92) |
| Not participating in any activity | 28,783 | 8,768 | 1.00 |  |  | 26,704 | 8,208 | 1.00 |  |
| Any activity | 24,867 | 7,924 | 1.01 | (0.96,1.06) |  | 23,067 | 7,445 | 1.01 | (0.96,1.07) |
