# Supplementary material for: Social influences on smoking cessation in mid-life: Prospective cohort of UK women
Source: PLoS One. 2019 Dec 6;14(12):e0226019. doi: 10.1371/journal.pone.0226019 (PMC6897408; doi:10.1371/journal.pone.0226019)
Supplement: S4 Table — (DOCX) [file pone.0226019.s004.docx]

# S4 Table. Sensitivity analysis for Figure 1, showing results adjusting for previous serious illness (hospital admission for heart disease, stroke, cancer, chronic obstructive airways disease) rather than self-rated health, and results after excluding women with previous serious illness

|  | **Adjusted for previous serious illness** | | | | |  | **Excluding previous serious illness** | | | | |
| --- | --- | --- | --- | --- | --- | --- | --- | --- | --- | --- | --- |
|  | **No. of current smokers** | **No. who ceased smoking in next 4 years** |  | **0R (95%Ci)** | |  | **No. of current smokers** | **No. who ceased smoking in next 4 years** |  | **OR (95% CI)** | |
|  |  |  |  |  |  |  |  |  |  |  |  |
| Partner is smoker at both time points | 10,333 | 2,014 |  | 1.00 | (1.00,1.00) |  | 9,587 | 1,881 |  | 1.00 | (1.00,1.00) |
| Partner is non-smoker at both time points | 17,867 | 6,113 |  | 2.01 | (1.86,2.17) |  | 16,746 | 5,790 |  | 2.02 | (1.87,2.19) |
| Partner is smoker at baseline and non-smoker 4 years later | 4,289 | 2,577 |  | 6.00 | (5.40,6.66) |  | 4,035 | 2,418 |  | 5.92 | (5.32,6.60) |
| Partner is non-smoker at baseline and smoker 4 years later | 619 | 73 |  | 0.54 | (0.39,0.75) |  | 580 | 72 |  | 0.56 | (0.40,0.79) |
